# Radiofrequency ablation: mechanisms and clinical applications

**DOI:** 10.1002/mco2.746

**Published:** 2024-10-02

**Authors:** Jianhua Wu, Zhiyuan Zhou, Yuanwen Huang, Xinyue Deng, Siting Zheng, Shangwen He, Genjie Huang, Binghui Hu, Min Shi, Wangjun Liao, Na Huang

**Affiliations:** ^1^ Department of Oncology, Nanfang Hospital Southern Medical University Guangzhou Guangdong China; ^2^ Department of Respiratory and Critical Care Medicine Chronic Airways Diseases Laboratory, Nanfang Hospital, Southern Medical University Guangzhou Guangdong China

**Keywords:** colorectal cancer liver metastases, combination therapies, hepatocellular carcinoma, incomplete radiofrequency ablation, tumor microenvironment

## Abstract

Radiofrequency ablation (RFA), a form of thermal ablation, employs localized heat to induce protein denaturation in tissue cells, resulting in cell death. It has emerged as a viable treatment option for patients who are ineligible for surgery in various diseases, particularly liver cancer and other tumor‐related conditions. In addition to directly eliminating tumor cells, RFA also induces alterations in the infiltrating cells within the tumor microenvironment (TME), which can significantly impact treatment outcomes. Moreover, incomplete RFA (iRFA) may lead to tumor recurrence and metastasis. The current challenge is to enhance the efficacy of RFA by elucidating its underlying mechanisms. This review discusses the clinical applications of RFA in treating various diseases and the mechanisms that contribute to the survival and invasion of tumor cells following iRFA, including the roles of heat shock proteins, hypoxia, and autophagy. Additionally, we analyze‌ the changes occurring in infiltrating cells within the TME after iRFA. Finally, we provide a comprehensive summary of clinical trials involving RFA in conjunction with other treatment modalities in the field of cancer therapy, aiming to offer novel insights and references for improving the effectiveness of RFA.

## Graphical abstract

Radiofrequency ablation (RFA) is a type of thermal ablation that induces coagulation necrosis of tumors by raising temperatures above 60°C. However, compared with complete RFA, because the ablation area cannot completely cover the entire tumor, insufficient RFA (iRFA) can lead to rapid local tumor progression, metastasis, and even further malignant transformation. There are three main causes of iRFA, including large or irregular tumor shape, heat sink effect, and adjacent organs/surfaces. In this review, we systematically summarize the underlying mechanisms contributing to tumor progression associated with iRFA, provide potential strategies to address the challenges posed by iRFA in order to enhance therapeutic efficacy.

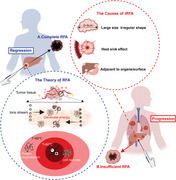

## 1 INTRODUCTION

**FIGURE 1:**
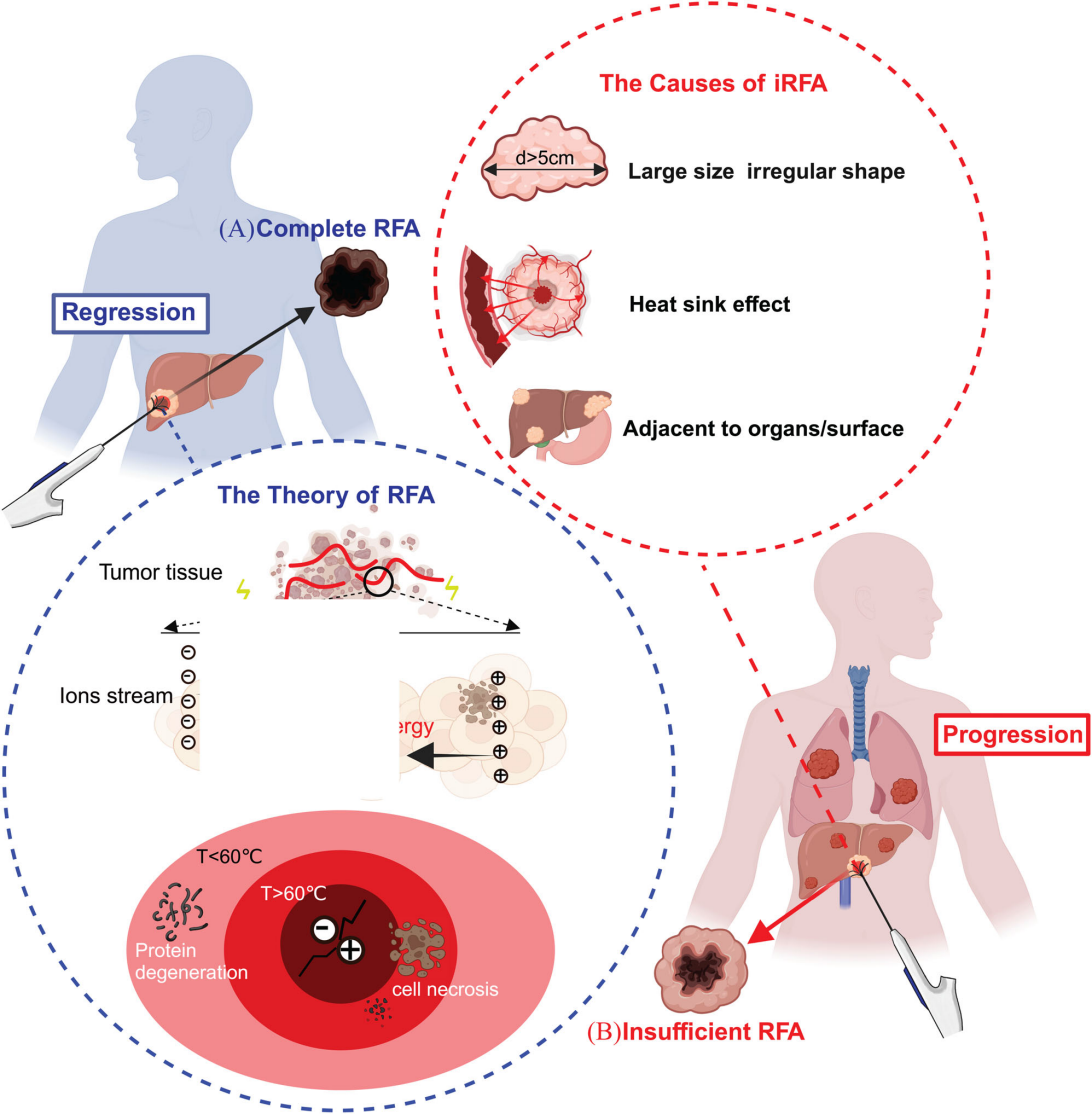
Therapeutic principles of RFA and main causes of iRFA. (A) Complete radiofrequency ablation: the theoretical basis for how RFA eliminates tumors: by inserting a radiofrequency needle into the tumor tissue and powering it on, the current causes ions in the tissue to vibrate and rub rapidly, generating heat. When the temperature reaches 60°C, proteins within the cells denature, causing cancer cells to dehydrate and degenerate, leading to coagulative necrosis and ultimately achieving the goal of destroying cancer cells. B: Insufficient Ablation: the causes of insufficient ablation: (1) large size and irregular shape; (2) heat sink effect; (3) adjacent to organs/surface.

Since the late 19th century, the discoveries made by physicist D'Arsonval regarding the induction of thermal energy in biological tissues through alternating radiofrequency ablation (RFA) have paved the way for the application of radiofrequency technology in disease management.^1^ Over the past century, RFA has been widely utilized in clinical practice for various conditions, including cardiovascular diseases, benign nodules, and tumors.^2^ RFA offers several advantages, such as minimal trauma and rapid recovery, making it an increasingly viable treatment option.^3^, ^4^ With ongoing advancements in imaging and RFA technology, this technique has demonstrated significant benefits in the treatment of tumors, particularly liver tumors.^5^, ^6^, ^7^ RFA is a form of thermal ablation that induces coagulation necrosis of tumors by raising temperatures above 60°C.^8^ The underlying principle of RFA involves inserting RF electrodes into tumor tissue and delivering RF current, which generates high temperatures within the tumor to achieve cell destruction.^9^ This process produces frictional heat by oscillating and agitating ions in tissues when exposed to high‐frequency alternating current (400–500 kHz).^10^, ^11^ The friction of ions in tissues generates heat, a phenomenon known as the Joule effect.^12^ Heat is then transferred outward from the thermal zone.^13^ Once the temperature reaches 60°C, intracellular proteins become denatured, and lipid bilayers melt.^14^ Consequently, tumor cells dehydrate and degenerate, leading to coagulative necrosis (Figure 1).

Although RFA has achieved significant success in various medical fields, it also has its limitations.^15^, ^16^ Currently, there is a lack of systematic reviews regarding the application of RFA technology. In this review, we systematically summarize the application of RFA across different diseases, clearly demonstrating its clinical spectrum. We particularly focus on the use of RFA in tumor treatment. From a mechanistic perspective, the threshold temperature and/or exposure time required to induce heat stress‐related cell death varies based on tissue and cell type.^17^ Therefore, cell‐type‐specific mechanisms may confer sensitivity or resistance to heat stress‐induced cell death.^17^, ^18^ Moreover, thermal ablation can destroy tumor vasculature and induce tissue ischemia and hypoxia, resulting in autophagy and apoptosis of tumor cells.^19^ Additionally, the large volumes of tumor fragments and biomolecules generated in situ, including tumor antigens and damage‐associated molecular patterns, can trigger an immune response that inhibits tumor growth.^20^ Overall, thermal damage from RFA contributes directly to tumor cell death through protein denaturation and may alter the tumor microenvironment (TME), mediating the antitumor immune effect. However, a major challenge associated with RFA is local recurrence, as well as the emergence of new intrahepatic and extrahepatic metastases following treatment in cancer patients.^21^ The ablation area often does not completely encompass the entire tumor, leaving residual tumor tissue after the procedure. This phenomenon is referred to as iRFA.^22^ While iRFA can partially reduce the size of the primary tumor, it may also lead to rapid local tumor progression, metastasis, and even further malignant transformation.^23^, ^24^, ^25^, ^26^

The primary causes for iRFA are outlined as follows (Figure 1): (1) The presence of large tumor size and irregular shape poses a significant challenge.^27^, ^28^, ^29^ Although RFA is effective for lesions measuring ≤3 cm in both hepatocellular carcinoma (HCC) and colorectal liver metastases (CRLM), achieving complete ablation for lesions exceeding 3 cm in diameter is relatively difficult.^30^ (2) The heat sink effect contributes to convective heat loss into adjacent blood vessels.^31^ Tumors located near blood vessels in the liver are more likely to experience iRFA and subsequent local recurrence.^32^ (3) The tumor's location is also a factor in the occurrence of iRFA, particularly when it is situated near the liver surface or vital organs, such as the stomach, intestines, and gallbladder.^33^ In these cases, the extent of ablation is often reduced to prevent damage, which can lead to iRFA. However, tumor ablation margins of less 5 mm are particularly susceptible to local recurrence.^34^ Overall, iRFA may lead to tumor recurrence due to residual tissue.^35^ Refer to Figure 1 for further details. Given that iRFA may inevitably occur in certain cases, further investigation into the molecular mechanisms that promote tumor progression will be beneficial for optimizing treatment timing, exploring combination therapy approaches, and enhancing the overall RFA treatment strategy.

In this review, we first begin with a systematic overview on the extensive clinical applications of RFA in existing diseases, with a particular focus on tumor research. We further analyze potential factors contributing to tumor progression associated with iRFA, including changes in the characteristics of residual tumor cells and the regulatory crosstalk within the TME. Finally, we propose potential strategies to address the challenges posed by iRFA in order to enhance therapeutic efficacy. By exploring the fundamental research on intracellular molecular mechanisms alongside clinical investigations of combined therapy models, it is hoped that potential targets to mitigate incomplete ablation‐induced tumor progression can be identified. This endeavor is expected to have significant implications for deepening theoretical knowledge about RFA, expanding its applications in the treatment of diseases, and improving therapeutic outcomes.

## 2 THERAPEUTIC APPLICATIONS OF RFA IN VARIOUS DISEASE CASES

### 2.1 Clinical applications of RFA in cancer treatment

According to the latest versions of the National Comprehensive Cancer Network and European Society for Medical Oncology guidelines for various cancers and treatment goals, the clinical applications of RFA in cancer therapy can be categorized into radical and palliative treatments (Table 1). Radical interventions are primarily used to manage precancerous lesions, early‐stage malignancies, or oligometastases. Palliative care reduces tumors, salvages postoperative therapy, or controls symptoms.

**TABLE 1:** Application in cancers.

Cancer type	Applied range	Technology of ablation mentioned in guideline	Treatment effect	Evidence level
**Therapeutic objective: radical treatments**				
Barrett's esophagus	Barrett's esophagus with LGD/HGD^36^	RFA (under endoscope)	Eradicate LGD and reduces the rate of progression from LGD to HGD and adenocarcinoma during 3‐year follow‐up^37^	NCCN: 2A
Esophageal and esophagogastric junction cancers	Patients with inoperable pTis, pT1a, pT1bN0, or stage pTis	Cryoablation or RFA (under endoscopic)	Lower incidence than surgical resection for early esophageal and esophagogastric junction cancers.	NCCN: 2A
	pT1a, pT1bN0 have residual dysplasia or Barrett's esophagus after surgery.^38^			
HCC	Patients with a single tumor ≤3 cm in diameter and well located^39^	Radiofrequency (or microwave) ablation	In small tumors <3 cm, RFA is as effective as resection in patients with early disease, with similar OS rate but a shorter procedure and hospital stay comparable to surgery.^5^, ^40^	NCCN: 2A ESMO: standard treatment
	Unresectable HCC^41^, ^42^, ^43^	Irreversible electroporation	In a small nonrandomized trial including 30 patients with malignant liver tumors, none of the eight patients with HCC experienced a recurrence through 6‐month follow‐up.^43^	NCCN: feasible choice
Hepatobiliary cancers	Same as HCC	RFA, cryoablation, percutaneous alcohol injection, microwave ablation	Survival rates for resection in patients who did not meet criteria for resection and receive other treatments were worse than those for ablation.^44^	NCCN: 2A ESMO: standard treatment
Kidney cancer	At high risk of surgery, isolated renal function, impaired renal function, hereditary renal cell carcinoma, or multiple bilateral tumors^45^	Thermal ablation (cryotherapy, microwave, radiofrequency) irreversible electroporation	It is recommended for inoperable patients with tumors <4 cm.^46^	NCCN: 2A ESMO: reasonable choice
	Stage T1 and stage T1b that are not suitable for or refuse surgical treatment^46^			
**Therapeutic objective: radical treatments + palliative treatments**				
NSCLC	Stage IA NSCLC^47^	Image‐guided thermal ablation therapy (cryotherapy, microwave, radiofrequency)	Ablation is an option for selected patients who do not receive SBRT or eventual radiotherapy.^47^	NCCN: 2A
	Local recurrence of local chest disease^48^			
	Multiple lung cancer			
Biliary tract cancers	Patients with recurrent or primary small single tumors <3 cm^49^	Thermal ablation (cryotherapy, microwave, radiofrequency)	complete ablation rate of 93% and a median OS of 30.2 months.^50^ Ablation can therefore be considered in patients with an ICCA <3 cm who have contraindications to surgery.^51^	NCCN: 2A ESMO: II, D
Central nervous system cancers	Patients with spinal metastasis^52^	Thermal ablation (cryotherapy, microwave, radiofrequency) radioablation	RFA of thoracolumbar and bone cement can control pain and health‐related quality of life^52^	NCCN: 2A
Uterine neoplasms	Additional ovarian ablation surgery is required for nonmenopausal patients after surgery.	RFA	Not mentioned.	NCCN: 2A
	Resectable isolated metastases^53^			
**Therapeutic objective: palliative treatments**				
Colorectal cancer	Oligometastases of the liver or lung that are not suitable for resection^54^, ^55^	Image‐guided thermal ablation therapy (cryotherapy, microwave, radiofrequency)	Lung ablation in colorectal cancer: OS at 5 years after surgery ranges from 40.7 to 67.5%.^55^	NCCN: 2A ESMO: III, B
			Provide good results for selected patients with small liver metastases with sufficient margins^56^	
Melanoma: uveal	Distant metastasis: isolated liver metastasis^57^	RFA	RFA can be used to treat liver metastases, and RFA ± liver surgery and liver surgery alone show similar OS and DFS.^57^	NCCN: 2A
Neuroendocrine tumors	Localized stage I–II lung metastasis with contraindications to surgery	Image‐guided thermal ablation therapy (cryotherapy, microwave, radiofrequency)	Liver metastasis resection + intraoperative ablation: OS was 80 and 59% at 5 and 10 years. Symptom‐free survival was 34% at 3 years and 16% at 5 years, independent of the tumor grade.^58^	NCCN: 2B ESMO: reasonable choice
	Locally advanced unresectable patients: ablation can be considered to add.^58^			
	Liver metastasis.^58^			
	Surgical contraindications for bronchopulmonary neuroendocrine tumors.			
Gastric cancer	Patients with gastric cancer associated hemorrhage	Not mentioned.	Short‐term control of gastric cancer related bleeding is good.	NCCN: 2A
Adrenal tumors	Advanced low‐metastatic adrenal cortical carcinoma and postoperative recurrent adrenal cortical carcinoma.^59^	Image‐guided thermal ablation therapy (cryotherapy, microwave, radiofrequency)	Not mentioned	ESMO: V, B
Soft tissue sarcoma	Stage IV patients with limited tumor volume	Image‐guided thermal ablation therapy (cryotherapy, microwave, radiofrequency) irreversible electroporation	Not mentioned	NCCN: 2A
	Single lesion or disseminated metastasis			
	Progressive, pathological, or symptomatic diseases^60^			
	Single liver and lung metastases^61^			
	Musculoskeletal metastasis			
Vaginal cancer	Patients with stage IVB or recurrent restrictive distant metastases	RFA, cryoablation	Not mentioned	NCCN: 2A
Mesothelioma: pleural	Patients with symptomatic pleural disease^62^	Image‐guided thermal ablation therapy (cryotherapy, microwave, radiofrequency)	Not mentioned	NCCN: 2A
Gastrointestinal stromal tumors	Patients with liver metastases after standard imatinib regimens^63^	Image‐guided thermal ablation therapy (cryotherapy, microwave, radiofrequency) irreversible electroporation	Not mentioned	NCCN: 2A
Thymomas and thymic carcinomas	Patients with inoperable or unresectable solitary or ipsilateral pleural metastases	Image‐guided thermal ablation therapy (cryotherapy, microwave, radiofrequency)	Not mentioned	NCCN: 2A
Bone cancer	Patients with inoperable lung metastases	Not mentioned	Not mentioned	NCCN: 2A
Thyroid carcinoma	Patients with symptoms or disease progression	RFA	Not mentioned.	NCCN: 2A
	Residual lesions or postoperative assistance after surgical resection.	RAI ablation, RFA		
	Local recurrence of limited lymph node load^64^	Ethanol ablation, RFA		
	Distant metastasis with symptoms	RFA, ethanol ablation, cryoablation		

Abbreviations: LGD, low‐grade dysplasia; HGD, high‐grade dysplasia; OS, overall survival; NSCLC, non‐small cell lung cancer; SBRT, stereotactic body radiotherapy; ICCA, intrahepatic cholangiocarcinoma; DFS, disease‐free survival.

#### 2.1.1 Radical treatments

RFA can be used for precancerous lesions, such as Barrett's esophagus. RFA alone may be useful for patients with Barrett's esophagus with confirmed low‐grade dysplasia (LGD) or high‐grade dysplasia (HGD). Previous studies have confirmed its safety and effectiveness in eradicating LGD and reducing the rate of progression from LGD to HGD and adenocarcinoma over 3 years of follow‐up.^36^, ^37^, ^65^ Additionally, patients with pTis, pT1a, and pT1bN0 esophageal cancer who are unable to undergo curative surgical resection may opt for ablation, which can also eliminate residual abnormal proliferation after esophagectomy.^38^ In HCC and cholangiocarcinoma cases, RFA achieves almost the same curative effects as surgery for lesions with a diameter of <3 cm at an appropriate location.^39^ Evidence shows that percutaneous RFA offers better OS and recurrence‐free survival (RFS) than surgical resection.^40^, ^66^ In other early‐stage cancer cases, RFA can effectively cure T1N0 non‐small cell lung cancer and has comparable efficacy to surgical resection.^67^ Patients with T1b kidney cancer who are elderly or in poor health and unsuitable for surgery may also undergo RFA or other thermal ablation techniques, often combined with multiple treatment modalities to achieve curative results.^45^, ^46^ RFA is an appropriate treatment option in early‐stage tumor cases and can lead to radical tumor cure. Furthermore, Table 1 shows that the use of RFA for treating oligometastases has been recommended in various guidelines involving neuroendocrine tumors, adrenal tumors, colorectal cancer liver metastases, lung metastases from soft tissue sarcomas, bone cancer lung metastases, solitary liver metastases from gastrointestinal stromal tumors, uterine tumors, isolated liver metastases from uveal melanoma, central nervous system tumors, pleural metastases from thymic carcinoma, and malignant pleural mesothelioma.

#### 2.1.2 Palliative care

RFA application can be broadly divided into three categories: (1) Tumor shrinkage. In cases of advanced‐stage primary lesions, RFA effectively reduces tumor size in cases of symptomatic advanced soft tissue sarcomas, bronchopulmonary neuroendocrine tumors contraindicated for surgery, limited volume stage IV soft tissue sarcomas, and symptomatic or progressing thyroid cancer. (2) Salvage option for recurrent primary lesions after radical surgery. RFA can also be used for residual lesions postsurgical resection or locally recurrent thyroid cancer cases with lymph node burden.^64^ (3) Ablation of bone and muscle metastases eliminates lesions and relieves pain symptoms.^52^ Endoscopic ablation can also be used to temporarily control bleeding associated with gastric cancer.

### 2.2 Clinical applications of RFA in treating other diseases

RFA has been widely used to treat non‐neoplastic diseases, including cardiovascular diseases, benign hemorrhagic gastrointestinal diseases, chronic painful osteoarthropathy, benign thyroid nodules, and others. The indications, efficacy, complications, and evidence‐based recommendations for using RFA to treat different diseases are summarized in Table 2. The following sections will provide detailed explanations for each aspect.

**TABLE 2:** Clinical applications of RFA in the treatment of other diseases.

Specific disease	Indication	Efficacy	Complication	Evidence	References
Cardiovascular disease					
AF	Symptomatic patients with recurrent paroxysmal or persistent AF resistant or intolerant to previous treatment with at least one Class I or III antiarrhythmic drug Symptomatic patients with recurrent paroxysmal AF Patients with AF and left ventricular systolic dysfunction, suspected to be related to arrhythmia‐mediated cardiomyopathy, to improve left ventricular function	A 53% elimination of AF at 1 year and a 99% reduction in AF burden^a^	Top three: asymptomatic acute cerebral lesions, 5–30%;vascular complications, 1–4% without ultrasound‐guided vascular puncture; cardiac tamponade, 0.4–1.3%	Expert consensus statement	^69^, ^87^
Ventricular arrhythmias	Most drug‐refractory ventricular arrhythmias	48% of patients were free of recurrent arrhythmia and another 19% were improved at the 6‐month follow‐up	A single pericardial effusion treated with percutaneous drainage and a left ventricular pacing lead dislodgement with no deaths in 31 patients	Prospective study (NCT01791543, NCT03204981)	^70^
Hypertrophic cardiomyopathy	Symptomatic drug‐refractory patients with obstructive hypertrophic cardiomyopathy	84.6% of patients achieved clinical success defined as noninvasive left ventricular outflow tract reduction >50% at 3 months postoperatively compared with baseline, symptoms in all patients improved and the median 6 min walking distance increased from 300 m at baseline to 556 m 3 months later without any safety endpoint events.	Only one patient presented with new‐onset right bundle branch block in 13 patients	Prospective study (ChiCTR2200066128)	^72^
Coronary artery fistula	Fistulas from coronary artery to pulmonary artery	Postprocedural narrowed of flow in the pulmonary artery and occlusive fistulas were observed, and the patient had no discomfort, with normal T waves and no abnormal flow from a coronary artery to the pulmonary artery at 3‐month follow‐up	Not mentioned	Case report	^73^
Cardiocutaneous fistula	Recalcitrant right ventricular fistula	Continuous fistulous drainage stopped without recurrence	Not mentioned	Case report	^74^
Brugada syndrome	Symptomatic patients with Brugada syndrome	No further ventricular tachycardia/ventricular fibrillation recurrences were documented after epicardial ablation during a median follow‐up of 10 months	Needle puncture of the right ventricle in ≈10% of patients, tamponade in ≈5%, and injury to abdominal viscera or coronary arteries in ≈1%	Prospective study	^75^
Gastrointestinal disease					
GAVE	GAVE	Endoscopic success 97% (95% CI, 79–100), the increase in hemoglobin level posttreatment 1.95 g/dL (95% CI, 1.62–2.27)	Bleeding ulcer (1.92%)	Systematic review and meta‐analysis	^77^
CRP	Hemorrhagic CRP	Rectal bleeding stopped completely in all patients during the mean follow‐up of 28 months (range 7–53 months). A significant improvement occurred in the mean (±SD) hemoglobin level from 11.8 ± 2 to 13.5 ± 1.6 g% (*p* < 0.0001).	Rectal discomfort and a burning sensation (12%). Transient fecal incontinence was reported in two patients of 39 patients.	Multicenter retrospective study	^78^
Hemorrhagic radiation esophagitis	Radiation esophagitis with recurrent upper gastrointestinal bleeding	A significant regression of the esophageal telangiectasias was observed in the 15‐month follow‐up. No recurrent bleeding occurred, and hemoglobin level remained stable.	Not mentioned	Case report	^79^
Intraductal bleeding	Hemostasis of an intraductal visible vessel	After two applications of RFA, cholangioscopy confirmed ablation and flattening of the vessel. No further bleeding has been found during 12 months of follow‐up.	Not mentioned	Case report	^80^
Duodenal mucosal reconstruction	Patient diagnosed with type 2 diabetes and was scheduled for duodenal mucosal reconstruction	Duodenal mucosa was ablated without bleeding or perforation. During 1 month follow‐up period, the patient stopped taking hypoglycemic drugs, fasting blood glucose decreased from 7.0 to 6.0 mmol/L (from 15.7 to 11.8 mmol/L 2 h after a meal), and glycosylated hemoglobin decreased from 7.1 to 6.2% without any discomfort.	Not mentioned	Case report	^81^
Osteoarthropathy					
Knee osteoarthritis	Patients with knee osteoarthritis	An improved pain relief at 4 weeks (WMD = −0.504; 95% CI: 0.708 to −0.300), at 12 weeks (WMD = −0.280; 95% CI: 0.476 to −0.084), at 24 weeks (WMD = −0.359; 95% CI: 0.573 to −0.144).	No serious adverse events were observed.	Meta‐analysis of randomized controlled trials	^82^
Facet joint pain	Low back pain from degenerative facet joints	At least 50% pain relief	Not mentioned	Systematic review	^84^
Thyroid disease					
Thyroid nodules	Benign nonfunctioning thyroid nodules	Therapeutic success was 97.8% and the absolute volume reduction of nodule volume was 80.3 ± 13.7% (range 38.7–100%) at the 12‐month follow‐up. Both mean symptom and cosmetic scores showed significant improvements.	The rate of major complications (transient voice change and hyperthyroidism) was 1.0% (three out of 276).	Prospective multicenter study	^85^
	Autonomously functioning thyroid nodules	Thyroid stimulating hormone normalization was achieved in 71.2% of patients and the volume reduction rate was 69.4% at a mean follow‐up period of 12.8 months.	No patients experienced hypothyroidism or a life‐threatening complication during follow‐up.	Systematic review and meta‐analysis	^88^

^a^ These data are from a randomized clinical trial (NCT01913522).

#### 2.2.1 Cardiovascular diseases

The two main applications of RFA in treating cardiovascular diseases are in cases of refractory tachyarrhythmias and hypertrophic cardiomyopathy, especially obstructive hypertrophic cardiomyopathy (oHCM).

In managing refractory rapid cardiac arrhythmias, RFA is considered a definitive treatment for several conditions: atrial fibrillation (AF) with lifestyle‐impairing symptoms and failure of at least one antiarrhythmic agent; symptomatic idiopathic ventricular tachycardia; symptomatic supraventricular tachycardia due to atrioventricular re‐entrant tachycardia, atrioventricular nodal re‐entrant tachycardia, unifocal atrial tachycardia, or atrial flutter.^68^ According to the guidelines, factors affecting rhythm outcomes, including age, duration of AF episodes, comorbidities, atrial dilation, and the presence of fibrosis, should be considered to assess whether catheter ablation is appropriate for patients with AF.^69^ The application and complications of RFA in cases of ventricular tachycardia are similar to those in AF cases. However, percutaneous access to the epicardial space is often limited for various reasons, leading to failure in ablating areas of ventricular arrhythmias. Stevenson et al.^70^ reported a multicenter series study involving 31 patients. They developed a radiofrequency needle injection ablation technique to identify and ablate deep endocardial arrhythmogenic substrates, showing promising prospects for future applications.^70^ Therefore, current research emphasizes the appropriate use of RFA in treating patients with refractory symptoms after failed drug therapy. However, the benefits of performing RFA on asymptomatic patients still require further evidence for confirmation.

In 2011, the first report of using RFA for 19 patients with oHCM with refractory symptoms and coronary artery anatomy unsuitable for alcohol septal ablation was published. The study achieved a 62% reduction in resting gradients, a 60% reduction of provoked gradients of the left ventricular outflow tract, and an improvement in exercise capacity and symptoms.^71^ Long et al.^72^ recently conducted a study on trans‐coronary radiofrequency ablation (TCRFA) for patients with oHCM, suggesting that TCRFA is a novel and effective nonsurgical treatment method focused on the coronary arteries. TCRFA produces more precise iatrogenic infarction around the radiofrequency wire than chemical ablation and is unaffected by anatomical variations in target interventricular septal perforator arteries. Significantly, selecting the optimal ablation strategy for cases of hypertrophic obstructive cardiomyopathy should be guided by a balanced analysis of the clinical benefits of reducing cardiac burden, the feasibility of achieving it, and the associated risks of complications. Patient preferences should also be considered in the shared decision‐making process.

RFA can also be used to treat some less common or rare cardiovascular diseases, such as coronary artery fistula,^73^ cardiocutaneous fistula,^74^ and Brugada syndrome.^75^ Its widespread application in treating cardiovascular diseases has made it an increasingly essential skill for cardiologists and vascular interventionists.

#### 2.2.2 Gastrointestinal diseases

In cases of gastrointestinal diseases, RFA is commonly used to ablate the gastrointestinal mucosa and effectively achieves hemostasis in several types of benign bleeding gastrointestinal diseases, such as gastric antral vascular ectasia (GAVE) and chronic radiation proctitis (CRP).^76^ The thermal approach of argon plasma coagulation (APC) is considered the first‐line endoscopic treatment for GAVE. However, a recent meta‐analysis including 24 studies involving APC (*n* = 508) and nine involving RFA (*n* = 104) suggests that the endoscopic and clinical success of RFA may indicate its potential role in managing patients with GAVE refractory to APC, leading to it eventually becoming the first‐line therapy.^76^, ^77^ Like GAVE, APC currently provides a cost‐effective endoscopic approach traditionally used by gastroenterologists. However, it has a high complication rate of approximately 47%, potentially leading to deep tissue injury, which results in ulcers, perforation, and fistula formation in patients.^76^ Rustagi et al.^78^ proposed that RFA therapy may avoid deep tissue injury and is a promising treatment for CRP. However, more controlled trials and studies are needed to compare the therapeutic effects, long‐term durability, cost effectiveness, and safety between these two intervention methods, and endoscopic RFA may potentially become the standard treatment for GAVE and CRP.

RFA is also used in hemostasis cases to treat hemorrhagic radiation esophagitis and intraductal bleeding.^79^, ^80^ A special application of RFA is reconstructing duodenal mucosa, which reduces intestinal glucose absorption by ablating the duodenal mucosa, thereby treating type 2 diabetes.^81^

#### 2.2.3 Osteoarthropathy

The thermal effect of RFA can also act on nerves and cause denervation of sensory nerve portions, thereby relieving intractable pain. A meta‐analysis of eight randomized controlled trials demonstrated that RFA achieved improved pain relief at 4 weeks (weighted mean difference [WMD] = −0.504; 95% confidence interval [CI]: 0.708 to −0.300), at 12 weeks (WMD = −0.280; 95% CI: 0.476 to −0.084), and at 24 weeks (WMD = −0.359; 95% CI: 0.573 to −0.144) with no serious adverse events in knee osteoarthritis treatment.^82^ The effectiveness of RFA in relieving facet joint pain has been recognized by consensus guidelines and systematic reviews from multidisciplinary working groups.^83^, ^84^ The application of RFA has currently been expanded to treat chronic pain conditions, such as degenerative small joint disease, sacroiliac joint pain, trigeminal neuralgia, chronic plantar fasciitis, and refractory shoulder pain.^82^

#### 2.2.4 Thyroid diseases

As a minimally invasive, safe, and effective alternative treatment option, RFA can also be used for the ablation therapy of benign, nonfunctioning thyroid nodules and autonomously functioning thyroid nodules (AFTNs). This approach has demonstrated satisfactory results in reducing nodule volume and improving symptoms such as dysphonia, hypocalcemia, and undesired cosmetic changes. In cases of benign nonfunctioning thyroid nodules, RFA achieved a therapeutic success rate of 97.8% and an absolute volume reduction of nodule volume at 80.3 ± 13.7% (range: 38.7−100%) at the 12‐month follow‐up, with significant improvements in mean symptom and cosmetic scores.^85^ In AFTN cases, RFA can be an alternative to radioactive iodine therapy, with the additional benefit of not causing hypothyroidism.^86^

In addition to the previously mentioned diseases, RFA is widely used in various other medical conditions. However, for the purpose of this paper, we will specifically focus on RFA in the context of tumors, particularly primary and metastatic liver tumors. The following discussion will delve into the specific regulatory mechanisms of iRFA, the alterations induced by iRFA on the tumor and its microenvironment, as well as contemporary treatment strategies that integrate RFA with other modalities.

## 3 ALTERATIONS AND REGULATORY MECHANISMS OF iRFA‐INDUCED TUMOR CELLS

Although RFA has demonstrated satisfactory clinical value, increasing evidence has demonstrated that residual tumor cells exhibit enhanced proliferative, metastatic, and metabolic adaptations under the therapeutic pressure of RFA. Next, we turn our attention to the specific mechanism regulation of insufficient ablation in liver tumors, desiring to find interventions to improve the efficacy of RFA. Tumor cells in a hyperthermic environment can be more resistant to higher temperatures than normal cells due to the obstructed activation of caspase 3.^89^ The human macrophage cells result in 100% death at 41° for 1 h.^89^ Therefore, there is a major problem in the hyperthermia treatment of patients with cancers/tumors because hyperthermia fails to induce apoptosis in cancer/tumor cells and may also damage more immune cells that inhabit the bloodstream in the process. Resistance and adaptability of tumor cells to high temperature expedite tumor progression and contribute to poor prognosis. The principal regulatory mechanisms include heat shock proteins (HSPs), hypoxia‐inducing factor (HIF), EMT, interleukin‐6 (IL‐6)/hepatocyte growth factor (HGF)/cellular‐mesenchymal to epithelial transition factor (c‐MET)/signal transducer and activator of transcription 3 (STAT3) signal axis, cancer stem cells (CSCs), epigenetic modifications, and autophagy. Based on the sequence of discovery of tumor‐related mechanistic evolution after RFA, we elaborate and present the alterations and regulatory mechanisms within tumor cells in a timeline manner. We also present and discuss the contents of the following subsections.

### 3.1 Heat shock proteins

**FIGURE 2:**
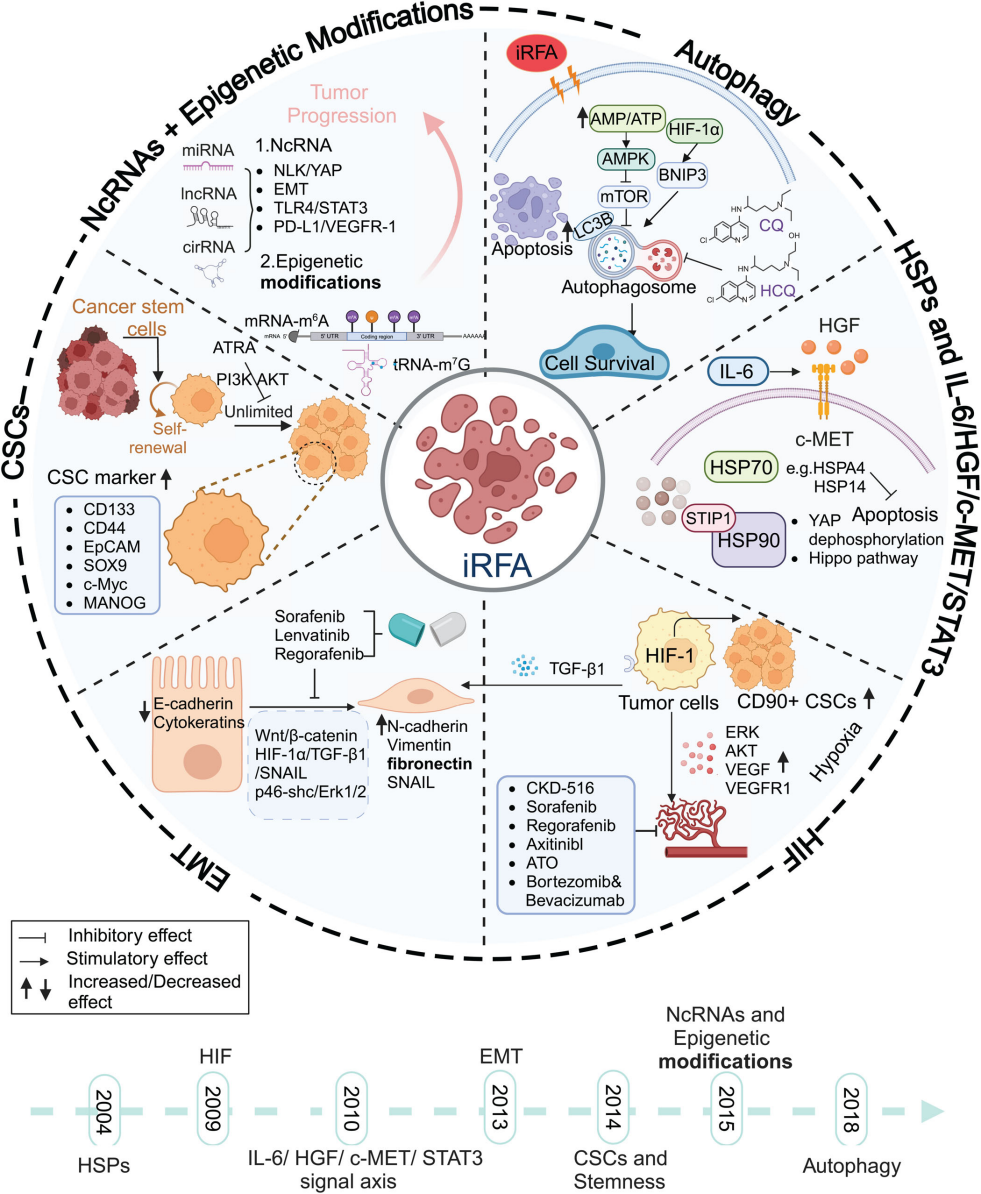
Alterations in tumor cell characteristics and their underlying regulatory mechanism following iRFA. In 2004, HSPs were first proposed to be associated with tumor recurrence and metastasis after iRFA. Subsequently, related research was carried out continuously. HIF‐VEGF, IL‐6/HGF/c‐MET/STAT3 signal axis, and EMT have also been suggested to be involved in tumor recurrence after iRFA. The increase in the dryness of tumor cells fuels the progression of residual tumor cells. Noncoding RNA and epigenetic modifications, as well as autophagy, also drive tumor progression. CQ, chloroquine; HCQ, hydroxychloroquine.

HSPs were one of the first factors that contributed to the recurrence of HCC/CRLM by iRFA in 2004.^90^, ^91^ HSPs are induced by various cellular stresses, including heat, hypoxia, injury, senescence, tumors, radiation, and cardiovascular diseases.^92^, ^93^, ^94^, ^95^ Heat stress‐induced protein denaturation and aggregation results in the upregulation of HSPs, a group of molecular chaperones with cytoprotective and antiapoptotic properties through the stress‐inducible transcription factor, heat shock factor 1 (HSF1). RFA causes focal hyperthermic injury to tumor cells, resulting in the upregulation of HSPs and the induction of thermal tolerance.^90^, ^96^, ^97^ Heat stress stimuli further increase the expression of HSPs, such as HSP27, HSP70,^91^ HSP90,^98^ and repress cell apoptosis by inhibiting proapoptotic factors, such as p53, Bcl‐2‐associated X‐protein, BH3 interacting‐domain death agonist, protein kinase B (AKT), attenuated familial adenomatous polyposis‐1 and other Bcl‐2 family members.^99^ Particularly, high expression of HSPA4 and HSPA14 (members of the HSP70s family) is associated with a poor prognosis^100^ due to its function in protecting mitotic cells against heat‐induced centrosome damage and division abnormalities.^101^ Normal cells arrest their cell cycle until centrosomal damage is repaired. However, tumor cells resume cell division before the centrosomes are repaired.^102^ Similarly, RFA enhances the expression of stress‐induced phosphoprotein 1, a cochaperone of HSP90 that promotes EMT and metastasis in hepatomas (Figure 2).^103^ Moreover, HSP90 inhibition induces apoptosis in HCC cells and decreases distant tumor growth.^104^, ^105^ With HSP90 involvement, heat shock induces YAP dephosphorylation and Hippo pathway activation,^106^ with clear evidence showing that this response enables the survival of mouse B16 melanoma cells following hyperthermia.^107^ In addition to the cytoplasmic response, HSF1 also initiates the unfolded protein response in the endoplasmic reticulum (ER) to assist in the protein folding capacity of ER.^108^ It also promotes the translation of prosurvival proteins’ mRNA, such as activating transcription factor 4.^109^ The peripheral heating zone adjacent to the central heating zone may not reach the lethal high temperature during RFA treatment, resulting in incomplete necrosis of tumor cells.^110^ The synthesis of HSPs in residual tumor cells is significantly increased upon exposure to high temperatures. The increased synthesis of HSPs helps protect tumor cells, leading to a higher recurrence rate and poorer prognosis.

Overall, these findings suggest that RFA enhances the expression of HSPs in tumor cells, thereby contributing to the malignant transformation of tumors. Therefore, targeted inhibition of HSPs may effectively reverse thermotolerance in cancer cells.^111^

### 3.2 Hypoxia‐inducing factor

Five years after HSPs was first identified as the mechanism through which iRFA leads to tumor recurrence, hypoxia has also been shown to promote HCC/CRLM progression after Irfa.^112^ Hypoxia is a key feature of the TME. Hypoxia‐inducing factor (HIF)‐1, a critical transcription factor, participates in the cellular response to hypoxia and plays a pivotal role in tumor development. As shown in Figure 2, iRFA can further disrupt tumor blood supply, exacerbate hypoxia, and activate HIFs within the TME.^113^ Studies have demonstrated that hypoxia contributes to increased metastasis, invasion, and drug resistance in HCC cells after sublethal heat treatment.^112^, ^114^ Hypoxia causes the autocrine activation of CD95 on colorectal tumor cells, thereby promoting local invasion and accelerated metastasis outgrowth in the hypoxic transition zone following RFA.^115^

Moreover, in response to hypoxia, post‐RFA tumor cells could upregulate the HIF‐1α/vascular endothelial growth factor (VEGF) signaling pathway to promote angiogenesis in the remaining liver cancer tissues.^116^ The overexpression of VEGF leads to the downstream activation of vascular endothelial growth factor receptor (VEGFR). VEGFR‐1, rather than VEGFR‐2, has been identified as a potential target for inhibiting post‐RFA angiogenesis.^117^ Therefore, targeting angiogenesis may be useful for reducing post‐RFA recurrence. For example, the combination of RFA and the vascular‐disrupting agent CKD‐516, which inhibits tubulin polymerization, could destabilize microtubules and compromise tumor vascularization.^118^ Arsenic trioxide can also inhibit the paracrine signaling of angiopoietin‐1 and angiopoietin‐2 by downregulating the expression of p‐Akt/HIF‐1α. This blocks angiogenesis in HCC cells after Irfa.^119^ Meanwhile, sorafenib, regorafenib, and axitinib have been documented to suppress the HIF‐1α/VEGFA pathway in hepatoma.^120^, ^121^, ^122^ In addition to single antiangiogenic drugs that inhibit the HIF–VEGF pathway, drug combinations, including bortezomib and bevacizumab, can have a similar effect.^123^ Therefore, these drugs may be effective in combination with RFA.

Tumor‐associated vascular endothelial cells (TAECs) are critical in the specific mechanism of hypoxic‐induced angiogenesis.^124^ They secrete various cytokines, including IL‐8, IL‐6, monocyte chemotactic protein‐1, and growth‐regulated oncogene α.^125^ iRFA significantly induces TAECs to upregulate the expression of these cytokines, thereby promoting HCC cell invasion.^113^ In contrast, HCC cell metastasis involves the entry of cancer cells into the lumen of blood vessels and their interaction with vascular endothelial cells.^113^ Intercellular adhesion molecule 1 (ICAM‐1), a major endothelial cell adhesion molecule (EpCAM), interacts with platelet glycoprotein IIb/IIIa to mediate platelet adhesion.^126^ After iRFA, ICAM‐1 in vascular endothelial cells activates platelets and increases endothelial permeability by downregulating vascular endothelial‐cadherin, damaging the endothelial monolayer barrier and promoting tumor cell metastasis.^127^

Overall, iRFA stimulates angiogenesis through the HIF‐1α/VEGF/VEGFR1 pathway, thereby accelerating tumor progression. Targeting the HIF–VEGF signaling axis and angiogenesis can be key to addressing rapid progression after RFA.

### 3.3 IL‐6/HGF/c‐MET/STAT3 signal axis

Furthermore, the involvement of IL‐6/HGF/c‐MET/STAT3 signal axis in this process was demonstrated in 2010.^128^ HGF and its receptor c‐MET are known to shape the hepatic microenvironment and promote tumor growth in liver tumors.^129^, ^130^ HGF and c‐MET levels are elevated in residual tumor cells following iRFA, along with their upstream regulator, IL‐6 (Figure 2).^131^ The most significant upregulation of IL‐6 was observed when RFA was performed at a temperature of 55°C.^128^, ^132^ Besides its role in promoting local tumor progression, iRFA activates the HGF/c‐MET/VEGF signal axis in distant tumors (Figure 2).^131^, ^133^ Driven by tumor‐derived HGF, tumor‐infiltrating T cells upregulated the expression of programmed cell death protein 1 (PD‐1) after RFA. This upregulation was reversed by administering sunitinib and sorafenib (Figure 2).^134^, ^135^ Several novel therapies have been developed to target this pathway, including the use of IL‐6 siRNA nanoparticles and combined inhibition of c‐MET and STAT3.^136^, ^137^ These therapies are promising for various application. These findings suggest that inhibiting the IL‐6/HGF/c‐MET axis may have significant potential in treating incomplete liver tumor ablation.

### 3.4 EMT

EMT was first proposed to be involved in iRFA‐mediated tumor progression in 2013.^138^ EMT activation is a pivotal event in tumor invasion and metastasis.^139^, ^140^ During this process, epithelial cells undergo phenotypic changes toward a mesenchymal state, resulting in enhanced cell motility and migration, particularly in residual tumor cells following iRFA (Figure 2). Exposure to heat stress induces the upregulation of EMT‐related transcription factors, including SNAIL in HepG2 and Huh7 cells.^141^, ^142^ Downstream mesenchymal markers, including vimentin and N‐cadherin, are significantly enhanced, whereas the epithelial marker E‐cadherin is downregulated after Irfa.^143^, ^144^ In addition, iRFA activates the Wnt/β‐catenin pathway to promote EMT in the residual hepatoma cells.^145^ Moreover, sublethal heat exposure enhances the phosphorylation of p46‐Shc, activating the downstream ERK1/2 pathway and EMT.^138^ This observation was consistent with other post‐iRFA fundings. HCC cells exhibit increased phosphorylation of Akt and ERK1/2.^143^ Importantly, Akt and ERK inhibition effectively reverses EMT, thereby significantly attenuating tumor cells’ invasive potential after iRFA.^143^ Similarly, sorafenib and lenvatinib have been demonstrated to suppress EMT by inhibiting the phosphorylation of Akt and ERK, suggesting their potential in preventing HCC progression after iRFA.^146^, ^147^ Moreover, the hypoxic TME was found to promote EMT through the HIF‐1α/TGF‐β1/SNAIL pathway after RFA.^19^

Based on these findings, iRFA contributes to EMT development in residual tumors. However, EMT, a classical biological characteristic of tumors, is regulated by multiple signaling pathways. Consequently, effectively identifying and targeting the key regulatory pathways responsible for EMT in remnant liver tumor cells remains a formidable challenge for future research.

### 3.5 CSCs and stemness

One year after discovering EMT, tumor stemness was first shown to be associated with iRFA.^148^ iRFA treatment induces the upregulation of tumor stemness in residual tumor cells, thereby expanding of the CSCs population (Figure 2). CSCs, representing a small subset of tumor cells with the ability to self‐renew and differentiate, play a pivotal role in diminishing tumor efficacy, promoting drug resistance, and driving disease progress.^149^, ^150^ Several surface markers and side population have been identified on liver CSCs, including EpCAM,^151^ CD133,^152^ CD44,^153^ CD13,^154^ CD90,^155^, ^156^ CD24,^157^, ^158^, ^159^ CD47,^160^ and OV6.^161^ The expression of the CSC markers CD44 and EpCAM was found to be increased in locally recurrent liver tumors after RFA, indicating poor prognosis.^148^ iRFA accelerates HCC recurrence by upregulating the CSCs' proportion of CD133 and EpCAM subsets and the expression of stem cell‐related genes. All‐trans retinoic acid (ATRA), an inducer of tumor stem cell differentiation, blocks the promotion of iRFA by inhibiting the phosphoinositide 3‐kinase (PI3K)/AKT pathway to trigger tumor‐initiating cell apoptosis.^162^ Therefore, the combination treatment of RFA and ATRA or AKT inhibitors is a promising potential strategy to improve the therapeutic efficiency of RFA.

To maintain their stemness characteristics, CSCs significantly upregulate the expression of stemness transcription factors, including SOX9, c‐Myc, and NANOG. Recent studies have demonstrated that SOX9 upregulation after iRFA can drive self‐renewal and tumorigenesis in HCC.^163^ Furthermore, iRFA activates the PKCα‐ERK1/2 pathway leading to the upregulation of Fra‐1, a well‐known regulator of c‐Myc transcription in CRLM.^164^ Moreover, multiple augmentations were observed in the CD133+ CSCs population after iRFA.^163^, ^165^ The HIFs mentioned above are also involved in the process. A persistent hypoxic environment is established within the liver tumor after iRFA treatment, resulting in an increased production of CSCs. However, the downregulation of HIF‐1α reduces their proportion.^19^ This suggests the involvement of the HIF‐1α pathway in regulating CSCs and promoting the secretion of TGF‐β1, which facilitates TGF‐β1‐dependent EMT in HCC cells. Ultimately, this cascade leads to recurrence and metastasis of HCC after RFA. Elevated levels of plasma VEGF, induced by iRFA, also upregulate CD133+ CSCs through a VEGFR2‐dependent mechanism by inducing NANOG expression.^166^ As a potent inducer of CSCs differentiation, ATRA effectively suppressed residual HCC growth after iRFA by eliminating CSCs via the PI3K/AKT pathway.^119^ Collectively, post‐iRFA liver cancer cells exhibit enhanced stemness due to dysregulated stemness transcription factors, thereby promoting tumor progression.

### 3.6 Noncoding RNAs and epigenetic modifications

Noncoding RNAs (ncRNAs) were shown to be associated with iRFA in 2015.^167^ MicroRNAs (miRNAs) and long ncRNAs (lncRNAs) are widely recognized for their roles in cancer pathogenesis, progression, and recurrence.^168^, ^169^ lncRNA and miRNA microarrays have been extensively used to explore the relationship between abnormal miRNA and lncRNA expression and post‐RFA recurrence.^170^ Among these, the expression of miR‐34a has been reported to be negatively associated with early recurrence, whereas higher expression of miR‐130b predicts worse prognoses.^140^, ^167^, ^171^ In addition to their role in predicting HCC recurrence, lncRNAs can serve as competing endogenous RNAs (ceRNAs) regulating miRNAs expression.^172^, ^173^, ^174^ For example, lncRNA GAS6‐AS2 promotes the malignancy of residual tumors after iRFA by functioning as a ceRNA for miR‐3619‐5p.^175^ The lncRNA ASMTL‐AS1 was found to be associated with the downstream activation of the NLK/YAP pathway by acting as a sponge for miR‐342‐3p. This exacerbates the malignancy of the residual liver cancer after iRFA.^176^ The lncRNA FUNDC2P4 was downregulated in Huh7 cells following in vitro thermal stimulation to simulate iRFA. This downregulation of lncRNA FUNDC2P4 promoted EMT, increasing tumor proliferation, invasion, and migration.^177^ The circRNA–miRNA–gene regulatory network plays a vital role in promoting residual tumor progression after iRFA. Examples include the circRNA/miRNA‐PD‐L1/VEGFR‐1^178^ and the circ‐BANP/Let‐7f‐5p miRNA/TLR4 pathway.^141^

Epigenetic modifications of DNA, RNA, and histone can also influence the recurrence and progression of HCC after iRFA (Figure 2). iRFA upregulates N6‐methyladenine (m6A) modification and increases its binding affinity to the reader protein YTHDF1. This interaction increases the translation of EGFR mRNA, ultimately promoting the proliferation and metastasis of HCC cells.^179^ METTL114‐mediated m6A modification induces the upregulation of E3‐ligase Nedd4 in residual HCC tissue after iRFA. This upregulation enhances TGF‐β/SMAD signaling and EMT, thereby promoting HCC recurrence and metastasis.^180^ tRNAs exhibit a wider range of modifications than those by mRNA.^181^ iRFA increased N7‐methylguanosine (m7G) tRNA modification mediated by methyltransferase 1 (METTL1) in residual HCC cells, promoting SLUG/SNAIL translation and, consequently, HCC metastasis.^182^ Moreover, the upregulation of METTL1 in HCC cells after iRFA enhances the translation of TGF‐β2, resulting in the formation of an immunosuppressive environment. This environment induces the production of CD11b+CD15+ polymorphonuclear myeloid‐derived suppressor cells (PMN‐MDSCs), reducing the infiltration of CD8+ T cells. These findings further support the progression of residual tumors.^183^

Increasing evidence links ncRNAs and epigenetic modifications to tumor progression following iRFA treatment. Therefore, exploring and elucidating the underlying mechanisms should be the focus of this field.

### 3.7 Autophagy

As a recent research hotspot in the field of oncology, autophagy was introduced in 2018 as a mechanism through which iRFA leads to tumor recurrence.^22^ Autophagy is a well‐established lysosomal degradation pathway and the primary cellular response to stress in eukaryotes.^184^ Figure 2 illustrates the diverse alterations in autophagy observed in tumor cells after iRFA. Interestingly, iRFA has been reported to significantly upregulate the expression of the autophagic marker LC3B, which was detected in the transition zone adjacent to the ablated tissue. This suggests that iRFA induces autophagy to sustain cancer cell proliferation.^22^, ^165^ The promotion of autophagy by iRFA may occur through the activation of the HIF‐1α/BNIP3 pathway.^185^ An increased cellular AMP/ATP ratio after iRFA can activate AMPK, which induces autophagy and inhibits apoptosis in tumor cells.^186^ It has been demonstrated that the upregulation of NOX4 can induce mitochondrial ROS production after iRFA, leading to mitophagy through Nrf2/PINK1 and promoting HCC cell survival.^187^ Furthermore, ROS also potentiates autophagy by activating Beclin‐1.^188^, ^189^ Accumulating evidence suggests that combining autophagy inhibitors including chloroquine and hydroxychloroquine with RFA may synergistically induce tumor apoptosis.^22^, ^186^ Therefore, coadministration of autophagy inhibitors with RFA can modulate and reverse the negative effects associated with iRFA.

Thermotherapy, which operates on similar therapeutic principles, has a broad spectrum of applications in tumor treatment. Apart from mechanisms that have been discussed, researchers have conducted extensive studies on how thermotherapy can promote tumor recurrence, including its effects on telomerase and DNA damage.^190^, ^191^

Telomerase is a unique ribonucleoprotein enzyme that adds telomeric repeats to the 3′ end of chromosomes and is crucial in telomere synthesis and DNA damage repair.^192^ Moderate hyperthermia (43°C) promotes human telomerase reverse transcriptase expression through HSPs and enhances telomerase activity in tumor cells, contributing to their survival.^190^, ^193^, ^194^, ^195^, ^196^, ^197^ Cancer cells are defective in DNA damage repair genes compared with normal cells, which impairs their ability to adequately deal with DNA damage. Consequently, cancer cells rely on upregulation of mutagenic pathways for DNA damage repair.^198^ Thermotherapy reportedly interferes with the DNA repair pathway by degrading the breast cancer gene 2 (BRCA2) protein, essential for repairing DNA double‐strand breaks through homologous recombination.^191^, ^199^, ^200^ Therefore, for BRCA2‐mutated tumors, the coapplication of PARP inhibitors (olaparib) during sublethal temperature stimulation inhibits the DNA damage repair function of tumor cells and enhances the antitumor effect.^201^, ^202^

Overall, iRFA or thermotherapy induces survival of residual tumor cells through multiple mechanisms. New research is continuing and it is hoped that relevant mechanisms or signaling pathways can be targeted to benefit patients in the future.

## 4 IMMUNOLOGICAL EFFECTS OF RFA‐INDUCED TME IN CANCER

Under iRFA pressure, in addition to the adaptive changes in tumor cells, the changes in infiltrating cells in the TME may play a more important role in the process of iRFA promoting tumor progression. As previously mentioned, releasing immunogenic cellular components triggers an inflammatory environment in the tumor following RFA. RFA can activate the immune response and promote the infiltration of various relevant cells, including antigen‐presenting cells (APCs) such as dendritic cells (DCs), and tumor‐killing cells such as CD8+ T and natural killer (NK) cells, to exert antitumor efficacy. However, this initial immune response has limited effectiveness in improving patient outcomes. As tumor‐killing cells continue to be activated, depletion markers on their surfaces are upregulated, leading to immune evasion by the tumor. Simultaneously, other infiltrating cells in the TME, such as tumor‐infiltrating lymphocytes (TILs), MDSCs, neutrophils, NK cells, macrophages, and DC cells also contribute to tumor invasion and metastasis, thereby promoting tumor progression. The bidirectional immune effects of RFA on the TME are systematically described below.

### 4.1 Increased antigen‐presenting function is accompanied by an enhanced tumor‐killing effect

#### 4.1.1 Dendritic cells

As one of the most powerful APCs, DCs focus on activating tumor‐specific T‐cell responses.^203^ Previous studies have demonstrated that iRFA enhances antitumor immunity by regulating DCs. Specifically, it promotes the local infiltration of DCs in melanoma cases.^204^ In a urothelial carcinoma model, iRFA also increased the DCs infiltration.^205^ Peripheral blood tests of HCC patients after RFA suggest that RFA may activate myeloid DCs by upregulating the levels of proinflammatory cytokines, including tumor necrosis factor (TNF)‐α and IL‐1b, thereby activating CD4+ T cells.^206^ Additionally, it includes the induction of DCs differentiation. Studies have shown that post‐RFA tumor lysate can induce the differentiation of monocyte‐derived DCs and enhance their antitumor effects.^207^

New strategies have been developed to enhance antigen presentation in DCs. OK‐432 stimulates DCs before adoptive transfer, resulting in tumor suppression when combined with RFA.^208^ ECI301, which is a derivative of CC chemokine ligand 3, recruits CCR1+CD11c+ DCs to RFA‐treated tumors, thereby augmenting the immune response against tumors.^209^ Furthermore, the optimal priming for DC vaccination before RFA is crucial for boosting antigen‐specific T‐cell responses and preventing cancer recurrence.^204^ These promising studies invariably confirm that the therapeutic efficacy of RFA can be promoted by stimulating the antigen‐presenting function of DCs in the TME.

#### 4.1.2 T cells

**FIGURE 3:**
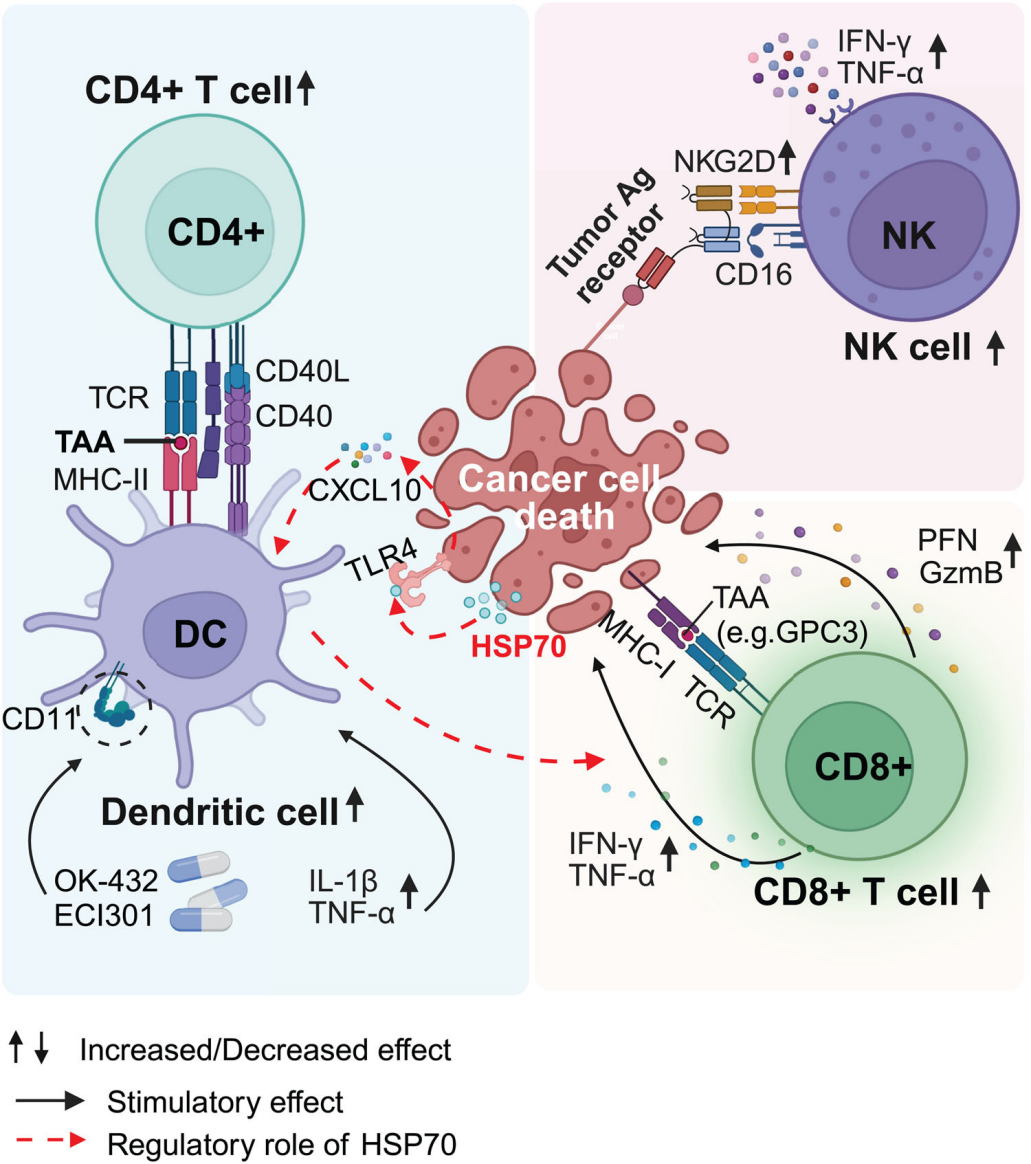
Mechanism of antitumor immune TME formation after RFA. RFA can activate the immune response and promote the infiltration of various relevant cells, including APCs (e.g., DCs), tumor‐killing cells (e.g., CD8+ T and NK cells), and “danger” signals (e.g., HSP70), to exert antitumor efficacy.

RFA can regulate various T‐cell functions, including antigen recognition, infiltration, and T‐cells cytotoxicity in liver cancer.^210^, ^211^ As mentioned above, RFA led to the activation of APCs, which increased the infiltration of CD8+ T cells, and to a lesser extent, CD4+ T cells in HCC and CRLM (Figure 3).^212^ Peripheral blood and tumor samples from liver tumor patients also showed a significant increase in the cytotoxicity of CD8+ T cells, creating a thermal tumor immune microenvironment and promoting antitumor efficacy.^212^ This is mainly related to the promotion of tumor‐associated antigen (TAA) release. T‐cell response to antigenic peptides from TAA was measured using interferon‐gamma (IFN‐γ) ELISPOT. More than 60% of patients who underwent RFA showed an increase in TAA‐specific T cells.^210^ The study demonstrated that RFA can increase in the number of T cells targeting Glypican‐3, one of the TAAs, in patients with HCC.^213^

RFA upregulates the expression of HSPs in tumor cells. HSPs can then act as T‐cell regulators and indirectly exert antitumor functions (Figure 3). Specifically, after treatment with transcranial arterial embolization combined with RFA, the expression level of HSP70 was positively correlated with CD8+ T cell infiltration around residual liver tumors.^214^ The HSPs‐tumor antigen peptide binding complex can be used as a foreign antigen to induce APCs, such as DCs, to cross‐present protoantigens to CD8+ T cells.^215^, ^216^, ^217^ However, the specific mechanism by which HSPs activates DCs in iRFA‐treated liver tumors requires further investigation. However, RFA dually enhances the expression of homing molecules on tumor‐draining lymph node (TdLN) high endothelial venules (HEV) and tumor vessels, promoting CD8 T‐cell trafficking across tumor vessels and HEVs in TdLNs.^218^ Therefore, in addition to its role as a palliative therapeutic modality, RFA may have clinical potential as an immune‐adjuvant therapy by augmenting the efficacy of adoptive T‐cell therapy.

Therefore, RFA has clear beneficial immunological results in increasing T‐cell transport or improving its efficiency. This effect is not sufficient for controlling HCC; however, it may represent the basis for developing an adjuvant immunotherapy in patients undergoing RFA for primary and secondary liver tumors.

#### 4.1.3 NK cells

In addition to the cytotoxic T cells that kill tumor cells, the antitumor effect of NK cells on RNA deserves great attention. NK cell‐mediated tumor surveillance and control can predict recurrence in post‐RFA HCC patients (Figure 3).^219^ Patient‐derived peripheral blood samples also suggested that a low level of IFN‐γ+ NK cells was correlated with an increased risk of recurrence.^220^ NK group 2D (NKG2D), which is an activated NK cells receptor, was upregulated after RFA treatment. This led to an increase in the total count of NK cells, and the production of IFN‐γ and TNF‐α in these cells, suggesting that RFA treatment enhances the antitumor activity mediated by NK cells.^221^ Mouse lung tumor models have also demonstrated that RFA treatment significantly increases the infiltration of macrophages, DCs, and NK cells in tumors, thus stimulating antitumor immunity.^222^ Therefore, promoting the antitumor activity of NK cells is expected to improve the efficacy of RFA.

Overall, these results suggest that RFA enhances the infiltration of immune cells involved in tumor killing and increases the expression of TAAs, including HSPs, in the TME. The activation of the antitumor immune response exerts tumor‐killing effects.

### 4.2 Immune response elicited by RFA negatively affects the effectiveness of liver cancer treatment

#### 4.2.1 TILs and MDSCs

**FIGURE 4:**
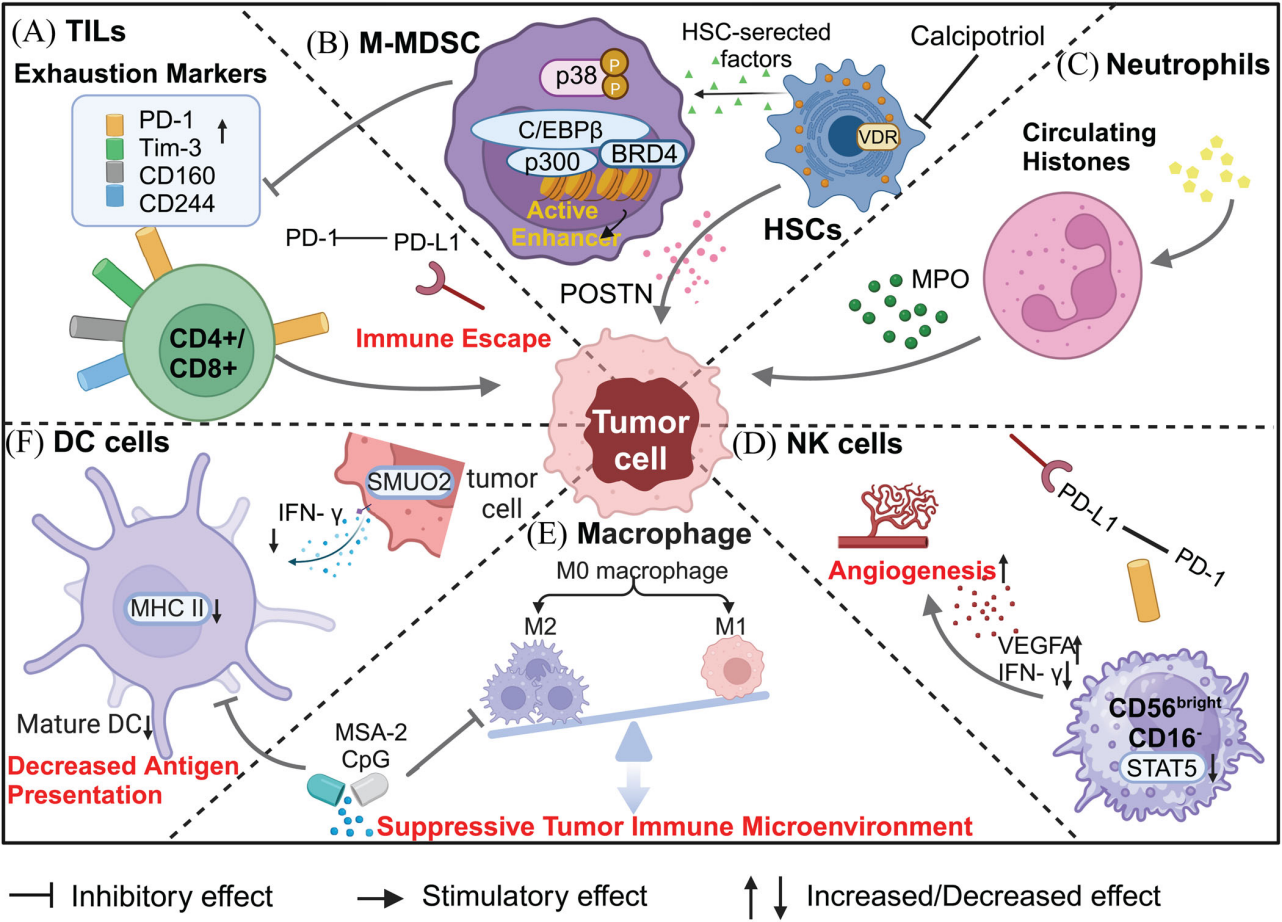
Mechanisms underlying the formation of an immunosuppressive microenvironment following iRFA. (A) Exhaustion markers (such as PD‐1, Tim‐3, CD160, and CD244) on TILs were upregulated after iRFA, ultimately resulting in immune evasion and malignant progression. (B) MDSCs are involved in inhibiting the antitumor immune response of TILs. (C) Elevated levels of histones in the peripheral blood stimulate neutrophils to produce cytokines, which in turn leads to the release of MPO. (D) The CD56^bright^CD16‐ NK cell suppresses STAT5 expression and upregulates VEGFA, thereby promoting angiogenesis. (E) M2‐like polarization of macrophages is involved in the formation of a suppressive tumor immune microenvironment. (F) Reduced maturation of DC cells participate in the attenuation of antigen‐presenting functions. POSTN, periostin; MPO, myeloperoxidase; MCP‐1, monocyte chemotactic protein‐1.

Despite the early activation of T‐cells following RFA, their functionality is impaired in later stages owing to alterations in memory T‐cell profiles and inadequate induction of long‐lived T‐cells by iRFA (after 24 weeks).^210^ Meanwhile, the expression of PD‐L1 on the surface of tumor cells also increased in CRLM patients after RFA treatment, concomitant with a gradual decrease in the antitumor immune response due to the upregulation of exhaustion markers (such as PD‐1, Tim‐3, CD160, and CD244) on TILs, ultimately leading to immune evasion and tumor progression (Figure 4A).^223^

As a crucial component of the hepatic TME, intratumoral MDSCs are upregulated after palliative RF.^224^, ^225^ MDSCs can promote HCC progression by interacting with hepatic stellate cells (HSCs) (Figure 4B). Activated HSCs can release factors that activate the p38MAPK pathway. This, in turn, activates C/EBPβ and P300, leading to BRD4 binding on enhancers. This process is accompanied by an increase in enhancer RNA and the specific presence of M‐MDSCs in monocytes. These events trigger enhancer reprogramming, and promote M‐MDSC differentiation and immunosuppression. The accumulation of M‐MDSCs is also associated with a decrease in cytotoxic T cells and HCC progression.^226^

Previous studies have demonstrated that RFA transiently activates innate and adaptive immunity but cannot sufficiently prevent cancer progression.^223^, ^227^ There are several potential mechanisms underlying the failure of the RFA‐induced immune response. First, residual tumors hinder the immune response by recruiting immunosuppressive myeloid cells to the TME.^25^ Moreover, tumor‐infiltrating T cells lose their effector functions and are exhausted shortly after iRFA, resulting in rapid tumor relapse.^223^ Therefore, a combination of immune checkpoint inhibitors and RFA has emerged as a promising approach for treating post‐RFA local recurrences. We believe that focusing on and elucidating the specific mechanisms of TME‐infiltrating cells and their interregulatory effects will be the key to improving RFA efficacy in the future.

#### 4.2.2 Neutrophils and NK cells

Neutrophils, one of the major components of the TME, exhibit contrasting antitumor or protumor roles at different stages of tumor progression. RFA‐induced necrosis has been found to increase the histone levels in the peripheral blood from HCC patients. This activation leads to cytokine production by the neutrophils, resulting in the release of myeloperoxidase. Consequently, this process promotes systemic inflammatory damage and HCC progression (Figure 4C).^228^, ^229^ Additionally, an elevated neutrophil‐to‐lymphocyte ratio (NLR) may predict poor survival in patients with CRLM after RFA.^230^ These studies suggest that neutrophils may promote tumor progression during iRFA. Neutrophils in the TME have been shown to exhibit significant heterogeneity and plasticity. Our study found that, residual tumor cells in CRLM undergo lipid metabolic adaptation after iRFA, which leads to increased infiltration of CD177^hi^PAD4^hi^ neutrophils, mediated by CXCL5‐CXCR1/2, and the release of NETs to promote tumor metastasis (unpublished data). Treatments targeting NETs reduce the incidence of tumor metastasis after iRFA. Therefore, the role of neutrophils in iRFA requires further investigation. Therapeutic strategies targeting neutrophils or NETs are likely to be valuable for the salvage treatment of iRFA.

The percentage of CD56^bright^ immature NK cells at a later time point (i.e., 1 month after RFA) was identified as a novel risk factor for post‐RFA recurrence.^231^ Tumor‐infiltrating NK cells that interact with PD‐L1 under hypoxic conditions are more prone to acquiring a CD56^bright^CD16^‐^phenotype, which inhibits STAT5 expression and leads to elevated levels of the angiogenic factor VEGFA, promoting angiogenesis (Figure 4D). Additionally, this phenotype reduces release of IFN‐γ, thereby compromising NK cells’ tumor cell‐killing activity.^232^, ^233^ These findings provide a new molecular perspective on the immunosuppressive effect of RFA therapy, suggesting the possibility of enhancing therapeutic efficiency and reducing tumor recurrence through the combined use of NK cell activators (such as anti‐KIR and SLAMF7‐targeting antibodies) and PD‐L1 monoclonal antibodies (e.g., durvalumab) during RFA therapy.^234^, ^235^, ^236^

#### 4.2.3 Macrophages and DC cells

iRFA accelerated the progression of the HCC residual tumor through macrophages M2‐like polarization.^25^ Furthermore, iRFA induces a suppressive tumor immune microenvironment, as evidenced by the promotion of macrophage M2‐like polarization and inhibition of antigen presentation by DCs, ultimately leading to the absence of intratumoral effector T cells and residual tumor progression (Figure 4E).^237^ The STING agonist MSA‐2 could reorganize M2‐like tumor‐promoting macrophages into M1‐like antitumor status and enhance the antigen‐presenting function of DCs. Therefore, the combination of MSA‐2 and RFA may activate antitumor immunity by reversing the iRFA‐induced immunosuppressive microenvironment, ultimately eliminating residual HCC tumors and reducing the postoperative recurrence rate. Similarly, administering the Toll‐like receptor‐9 agonist, cytidine phosphate guanosine (CpG), to DCs phagocytosing 65°C‐treated EG7 cells enhances the expression of major histocompatibility complex class II on DCs and the frequencies of M1 macrophages, leading to enhanced cytotoxic T lymphocytes (CTLs) responses and the potent inhibition of iRFA‐treated tumor growth.^238^

In addition, the sublethal heat stimulation induced by iRFA may inhibit the IFN‐1 signal in HCC by activating small ubiquitin‐like modifier 2 (SUMO2)‐mediated SUMOylation, potentially inhibiting DC cell maturation and CD8+T cell infiltration (Figure 4F).^239^ Therefore, targeting SUMOylation might enhance immune surveillance and offer a potential avenue to prevent HCC recurrence post‐RFA treatment.

In addition to tumor and immune cells, some normal tissue cells, such as liver‐resident cells, are also involved in the mechanism of iRFA‐mediated liver tumor recurrence. Liver‐resident cells, such as Kupffer cells and HSCs, create a distinctive microenvironment that influences tumor progression following iRFA.^240^ iRFA upregulates the expression of proteinase 3 in Kupffer cells, which promotes the growth of residual HCC through multiple oncogenes and the PI3K/AKT and P38/ERK signaling pathways.^241^ There are also several studies on HSCs that suggest a negative impact. For example, HSCs release periostin to promote the malignant phenotype of residual tumor cells through integrin β1 and p52Shc‐ERK1/2 pathways. This process involves stimulating the proliferation and metastasis of HCC cells by triggering EMT and decreasing the apoptosis of residual HCC cells after iRFA.^242^

Notably, various cell types in the TME perform distinct functions and roles when exposed to RFA stress. However, further research is necessary to unveil the enigmatic nature of how iRFA promotes tumor progression and to identify potential mechanisms and targets for enhancing its effectiveness.

## 5 THERAPEUTIC STRATEGIES COMBINED WITH RFA

Many patients experience rapid progression after RFA. Numerous studies have investigated the causes of this phenomenon, described in detail in the previous section. Several clinical trials on RFA combination therapy have addressed this issue. We searched the clinicaltrials.gov website and PubMed to identify all clinical trials involving RFA combination therapy for HCC and CRLM. We extracted prospective clinical trials that were either recruiting or completed, and focused on experimental protocols comparing RFA combination therapy to RFA‐only control and single‐arm trials of RFA combination therapy. The following two categories were identified based on the type of combination therapy strategy: RFA combined with local therapy (Table 3) and RFA combined with systemic therapy (Table 4).

**TABLE 3:** Local therapy combination with RFA.

Combination type	Start year	Region	Schedule	Current status	Phase	Ref. or trial ID	Primary outcomes/results	Disease type
Interventional therapy+RFA	2013	China‐ Taiwan	RFA+TACE vs. RFA	Unknown	II	NCT01858207	The rate of complete necrosis	HCC
	2008	China	RFA+TACE vs. RFA	Unknown	N/A	NCT00730860	disease free survival	HCC
	2008	China	RFA+TACE vs. RFA	Completed	Randomized and controlled	NCT00554905 PMID23269991	Overall Survival The 1‐, 3‐, and 4‐year overall survivals: 92.6, 66.6, 61.8% vs. 85.3, 59, 45.0% (*p* = 0.002)	HCC
	2007	China	RFA+TACE vs. RFA	Unknown	Randomized and controlled	NCT00556803	Overall survivals	HCC
	2003	Japan	RFA+TACE vs. RFA	Completed	N/A	PMID19567647	The 1‐, 2‐, 3‐, 4‐year local tumor progression rates: 14.4, 17.6, 17.6, 17.6% vs. 11.4, 14.4, 14.4, 14.4% (*p* = 0.797).	HCC
	2002	China	RFA+TACE vs. RFA	Completed	Randomized and controlled	NCT01415063 PMID22157201	Overall Survival 1‐, 3‐, 5‐years OS: 94, 69, 46% vs. 82, 47, 36% (*p* = 0.037)	HCC
	2000	Japan	RFA+TACE	Completed	N/A	PMID23068563	The 3‐, 5‐, 7‐year overall survival rates: 79.3, 60.6, 50.9%	HCC
	1996	USA	RFA+HAIC of floxuridine/5‐FU	Completed	II	NCT00004142 PMID12734081	At 20 months’ median follow‐up, 32% of patients remained disease free.	CRLM
PDT+RFA	2006	Global	Talaporfin sodium+RFA	Completed	III	NCT00355355	Overall survival	HCC
Radiotherapy+RFA	2019	China	RFA+ radiotherapy vs. RFA	Not yet recruiting	III	NCT03988998	2‐years recurrence rate	HCC
	2019	China	RFA+SBRT vs. RFA	Recruiting	III	NCT04202523	DFS	HCC
	2010	China	RFA+metuximab vs. RFA	Completed	II	PMID25210200	Overall tumor recurrence: 17 months vs. 10 months (*p* = 0.03)	HCC
	2008	China	RFA+radiotherapy vs. RFA	Unknown	Randomized	NCT00557024	Overall survivals	HCC
	2002	China	RFA‐125I vs. RFA	Completed	III	NCT01717729 PMID25064436	recurrence rate 1‐, 3‐, and 5‐years recurrence rate: 4.5, 22.1, 39.8% vs. 14.8, 35.3, 57.4% (*p* = 0.004)	HCC

Abbreviations: TACE, transcatheter arterial chemoembolization; HAIC, hepatic artery infusion chemotherapy; PDT, photodynamic therapy.

**TABLE 4:** Systematic therapy combination RFA.

Combination	Start year	Region	Schedule	Current status	Phase	Ref. or trial ID	Primary outcomes/results	Disease type
Chemotherapy+RFA	2010	China	ThermoDox+RFA	Completed	N/A	PMID31436231	The mean OS: 68.5 ± 7.2 months vs. 46.0 ± 10.6 months (*p* = 0.045).	HCC
	2008	Global	ThermoDox+RFA vs. RFA	Completed	III	NCT00617981 (HEAT) PMID29018051	Overall survival (fail to reach) OS HR: 0.95(95% CI, 0.76–1.20; *p* = 0.67)	HCC
Targeted therapy+RFA	2017	China	RFA+sorafenib vs. RFA	Unknown	N/A	NCT03097848	1‐year disease‐free survival	HCC
	2017	China	RFA+sorafenib vs. RFA	Unknown	N/A	NCT02187081	2‐year incidence of tumor recurrence	HCC
	2014	China	RFA+sorafenib vs. RFA	Unknown	N/A	NCT01470495 (REPEAT)	Time interval between new lesions emerging after the first HCC recurrence	HCC
	2010	China	RFA+sorafenib vs. RFA	COMPLETED	II	PMID25683938	Recurrence rate: 56.7 vs. 87.5% (*p* < 0.01).	HCC
	2009	USA	RFA+sorafenib vs. RFA	COMPLETED	II	NCT00813293 PMID34297268	Size of coagulation zone: 30.67 vs. 30.47 cm^3^ (*p* = 0.80)	HCC
Targeted therapy+immunotherapy+RFA	2022	China	RFA+regorafenib and toripalimab	Recruiting	II	NCT05485909	ORR	CRLM
	2022	China	RFA+tislelizumab/sintilimab+lenvatinib/bevacizumab vs. RFA	Recruiting	N/A	NCT05277675	1‐year recurrence‐free survival overall survival	HCC
	2022	China	RFA+toripalimab+lenvatinib	Not yet recruiting	N/A	NCT05162898	Recurrence‐free survival time	HCC
	2021	France	Atezolizumab+bevacizumab+RFA vs. RFA	Recruiting	II	NCT04727307	2‐year recurrence‐free survival	HCC
Immunotherapy+RFA	2019	Germany	RFA+pembrolizumab	Recruiting	II	NCT03753659	ORR	HCC
	2019	China	H101 (recombinant human adenovirus type 5)+RFA vs. RFA	Unknown	N/A	NCT03790059	Tumor‐free survival	HCC
	2016	China	RFA+highly purified CTL vs. RFA	Unknown	III	NCT02678013	Recurrence‐free survival	HCC
	2015	China	RFA+CIK vs. RFA	Completed	III	NCT02419677 PMID27855365	The 3‐year progression‐free rates: 20.3 vs. 13.3%	CRLM
	2009	Korea	RFA+DC vaccination vs. RFA	Completed	I/IIa	PMID26657650	TTP: 36.6 months (median) vs. 11.8 months (median) (*p* = 0.0031)	HCC
	2008	Korea	CIK cell agent (Immuncell‐LC)+RFA vs. RFA	Completed	III	NCT00699816 PMID25747273	RFS:44.0 vs. 30.0 months (*p* = 0.01)	HCC
Mutiple‐treatment +RFA	2019	China	Anlotinib+TACE+RFA	Recruiting	II	NCT04157140	TTP	HCC

Abbreviations: HEAT, a large global multicenter Phase III trials; CIK, cytokine‐induced killer; ORR, objective response rate; TTP, time to progression.

RFA combined with local therapy includes transcatheter arterial chemoembolization (TACE), hepatic artery infusion chemotherapy (HAIC), photodynamic therapy (PDT), and radiotherapy. Two randomized controlled trials conducted on RFA+TACE showed favorable outcomes. Patients with HCC underwent RFA and TACE had a significantly better OS than those who underwent RFA only. However, RFS differed between the two groups. The application of RFA+TACE in patients with HCC who have not received prior treatment can improve RFS; however, no such benefit was observed in patients with recurrent HCC after radical surgery.^243^, ^244^ A possible reason for this is that the tumor cells of patients with recurrent HCC, who have undergone radical surgery, may have spread to other areas beyond the targeted ablation region during surgery. Therefore, local treatment may not always effectively eradicate these cells.^245^ Furthermore, a trial of RFA combined with HAIC did not show any significant benefit to patients,^246^ and only one trial was found for RFA combined with PDT; however, no results were published (NCT00355355).

With the rapid technological advancements in radiotherapy, many clinical trials have explored the combination of RFA with radiotherapy. For example, two clinical trials investigating the use of intraradiation therapy combined with RFA produced significant findings. One trial demonstrated that percutaneous implantation of I125 particles in the liver, combined with RFA therapy significantly reduced intrahepatic and local recurrence rates in patients with HCC who were initially treated and did not have extrahepatic metastasis.^247^ Another study found that combining RFA with metuximab (a novel I131‐labeled monoclonal antibody used for guided radiation therapy in liver cancer) reduced the ORR in patients with HCC without extrahepatic metastasis.^248^ External beam radiotherapy is more commonly used in clinical practice than internal beam radiotherapy. As adjuvant treatment after surgery, external beam radiotherapy can prevent local recurrence and improve DFS in patients with suboptimal resection margins. This may also be beneficial in patients undergoing RFA.^249^ We anticipate the results of trials that combine external beam radiotherapy with RFA. Further, clinical studies should demonstrate the effectiveness of combining these local therapies as treatment modalities.

RFA combined with systemic therapy includes chemotherapy, targeted therapy, and immunotherapy. Although chemotherapy is the conventional treatment, few prospective trials have evaluated its efficacy in combination with iRFA for HCC or CRLM. A novel chemotherapeutic drug, thermosensitive liposomal doxorubicin (Thermodox), combined with RFA, showed promising results in a small‐sample clinical trial. However, no positive outcome was found in a large global multicenter Phase III trials (HEAT).^250^

Recent in‐depth research has shown that post‐iRFA progression is associated with the interaction between various components of the TME and the biological and behavioral changes in residual tumor cells. Therefore, recent clinical trials of RFA combined with systemic therapies have focused on targeted drugs and immunotherapy. The combination of sorafenib and RFA reduced the recurrence rate in previously untreated patients with medium‐sized (3.1−5.0 cm) HCC.^251^ Moreover, a small‐scale Phase II single‐center clinical trial demonstrated the effectiveness and safety of applying an adjuvant DC vaccine in patients who underwent surgical resection, RFA, percutaneous ethanol injection, or TACE.^252^ The same team conducted another multicenter Phase III clinical trial, demonstrating that postoperative adjuvant immune cell adoptive therapy significantly reduced recurrence rates and extended RFS.^253^ Recently, several clinical trials have recruited patients to study the effectiveness of targeted therapy, immunotherapy, and a combination of targeted‐immune therapies and RFA. Furthermore, another study is currently recruiting participants to investigate the efficacy of combining RFA with local and systemic therapies (RFA+TACE+anlotinib). Generally, with targeted drugs and immunotherapy becoming more prevalent in clinical practice, we anticipate future results that will help us redefine combination therapy strategies and enhance the effectiveness of RFA therapy.

Notably, these tables do not cover retrospective clinical trials. Some meaningful data may inform prospective trials on combination therapies. For example, a retrospective study showed that SBRT as a treatment for HCC after iRFA resulted in improved PFS and OS and lower rates of local disease progression than repeated RFA.^254^ Additionally, retrospective clinical data have shown that the combination of PD‐1 and RFA may enhance T‐cell immune response in patients with CRLM, resulting in an improved prognosis.^25^ However, whether patients can benefit from the combination of PD‐1 and RFA remains uncertain, which requires further research. Finally, most of the clinical trials listed in the tables were in the recruitment phase, with a significant number being Phase I and II clinical trials. This is particularly true for trials involving targeted therapy and immunotherapy combined with RFA. Currently, evidence from large‐scale, multicenter Phase III clinical trials is lacking; therefore, further investigation is needed for the strategy of combining therapies with RFA.

Besides, some new drug formulations or nanomaterials have been reported in preclinical studies for use in combination with RFA. For instance, the previously mentioned immunoenhancer OK432 can enhance the infiltration and function of DCs and CTLs within tumors, reduce the infiltration of regulatory T cells, and upregulate the levels of IFN‐γ and TNF‐α. It can also enhance the antigen‐presenting function of DCs by activating the cGAS–STING pathway, thereby improving antitumor immune effects.^208^, ^255^, ^256^, ^257^, ^258^ Nanomaterials mainly include the following three types: nano‐vaccines, novel carriers, and heat‐conductive media. Nano‐vaccines encompass LDHs–cGAMP, bisphosphonate nano‐vaccines (BNV), pH‐dependent HLCaP nano‐reactors, and ultra‐small metal TPZ composite Fe‐TPZ nanoparticles with deep tumor penetration. These nano‐vaccines can enhance the function and tumor infiltration of immune cells such as DCs, T cells, and macrophages, achieving a synergistic antitumor effect when combined with RFA.^259^, ^260^, ^261^ Besides, injecting heat‐conductive nanoparticles such as gold, copper, iron, or carbon around the RFA target area can enhance thermal transfer during the RFA process when a certain volumetric fraction is reached. This improves the efficiency of ablation by facilitating better heat distribution and transfer within the targeted tissue.^262^ However, research on these advanced drugs or materials is still at the cellular or animal level, and their pharmacokinetics and potential adverse effects are not yet well understood. This uncertainty may be a barrier to initiating clinical trials. We are looking forward to future studies exploring the combination of these novel drugs or materials with RFA in clinical settings, which could enhance the efficacy of RFA treatment.

## 6 CONCLUSION AND FUTURE PERSPECTIVES

In recent years, the application of RFA has increased significantly. However, its widespread utilization remains limited due to the phenomenon known as iRFA. A systematic review of landmark studies related to RFA could enhance our understanding of the role of iRFA in cancer recurrence and metastasis, thereby contributing to the development of innovative strategies to mitigate its disadvantages and achieve optimal clinical efficacy. In this review, we provide an overview of the current applications of RFA in various diseases, elucidating the definition and potential etiology of iRFA in malignant cases. We also comprehensively summarize the alterations within the tumor and its microenvironment induced by iRFA. Finally, we discuss contemporary treatment strategies that integrate RFA with other modalities and contemplate future directions for advancement.

The earliest documented heat application for tumor treatment can be traced back to Egyptian and early Greek medical practices, where cautery was used to treat superficial tumors.^263^ Malignant cells exhibit greater susceptibility to thermal injury than normal cells.^264^ Relevant studies have increasingly focused on applying RFA to treat primary and metastatic liver tumors, contributing to a growing body of research in this field.^265^, ^266^, ^267^, ^268^ The efficacy of RFA in inducing tumor necrosis and its therapeutic potential have been validated using imaging and pathological analyses.^10^, ^269^, ^270^ RFA has recently emerged as a prominent modality in treating malignant tumors, especially in cases of small liver cancer (<3 cm in diameter) and CRLM.^271^, ^272^, ^273^, ^274^ It represents a viable surgical alternative for achieving curative treatment and reducing complications.

However, the main challenge in the clinical application of RFA is achieving complete ablation to prevent residual tumors and, consequently, reduce tumor recurrence and metastasis. Therefore, current research primarily focuses on the following three directions. First, RFA technology is optimized by enhancing image navigation, upgrading ablation equipment, and refining ablation needles. For example, real‐time virtual sonography‐assisted RFA based on computed tomography (CT) or magnetic resonance images effectively treats conspicuous and inconspicuous HCC and hepatic metastases.^275^ Moreover, several studies have focused on the feasibility of real‐time in vivo assessment of RFA using spectral analysis, electrical impedance data, and machine learning models.^276^, ^277^, ^278^ Second, it is crucial to develop effective methods for the early detection and precise identification of iRFA. The initial research direction focused on imaging techniques such as contrast‐enhanced ultrasound, CT, and Positron emission tomography‐CT, which have a specific application value in detecting post‐RFA tumor residuals.^279^, ^280^, ^281^ However, imaging often struggles to detect small residual tumor lesions. With the advancement in liquid biopsy techniques, such as circulating tumor DNA (ctDNA), there is a growing emphasis on exploring the early detection of iRFA using ctDNA and other biomarkers.^282^ Third, it is imperative to investigate efficacious salvage therapies for cases of iRFA. This aspect will be addressed in subsequent sections. The solution to these problems will enhance the pivotal role of RFA in malignant tumor treatment.

In cases of non‐neoplastic diseases, RFA has also emerged as a feasible and secure approach and is widely used to treat cardiovascular diseases, benign hemorrhagic gastrointestinal diseases, hepatic hemangioma, chronic painful osteoarthropathy, and benign thyroid nodules. However, like many innovative technologies, more direct comparative trials, cost–benefit analyses, and long‐term follow‐up data are still required to determine the long‐term safety and efficacy of RFA in these applications. For instance, catheter ablation is used only for symptom management in cases of cardiovascular diseases. Therefore, whether asymptomatic patients and patients with AF and psychological distress can derive any benefits from it remains unclear.^283^ Notably, various factors still restrict the expansion of its application. In cases of gastrointestinal diseases, RFA may provide a deterministic, palliative, or alternative treatment for various gastrointestinal and hepatopancreatic biliary lesions. In contrast, the high costs associated with RFA may hinder its advancement.^76^ However, despite this, the indications for RFA continue to broaden with more extensive use and improved implementation.

Notably, several basic and clinical investigations have been conducted to explore the potential of RFA as a combination therapy in cancer treatment and the strategies for salvaging iRFA. Generally, RFA leads to the necrosis of tumor cells through heat damage. However, failure to fully encompass the tumor lesion will promote the proliferation and invasion of the remaining tumor cells, thereby accelerating tumor recurrence and metastasis. Accumulating evidence has explored the potential mechanisms underlying this phenomenon. As previously described, pathways associated with tumor progression, including angiogenesis and EMT, among others, are activated in residual tumor cells after iRFA. TME regulation remains an important factor in tumor progression after iRFA, due to T‐cell depletion and infiltration of immunosuppressive cells.

The TME evolution mediated by iRFA exhibits dualistic characteristics. For example, in the development of DCs and T cells, in the early stages of RFA, the increase in tumor neoantigens caused by thermal injury promotes antigen presentation by DCs, which recruits and activates T cells to perform antitumor functions.^204^, ^206^, ^212^ However, as time passes, the onset of iRFA inhibits the maturation of DCs and promotes the depletion of T cells, thereby converting the TME to an immunosuppressed state.^223^, ^239^ Overall, the mechanisms through which iRFA regulates tumor progression are diverse. Therefore, further exploration and study of potential mechanisms are needed to identify additional targets for clinical treatment.

Currently, several clinical trials have evaluated strategies to optimize the efficacy of RFA treatment. We reviewed the clinical trials conducted to date and found that most combination therapies are effective. Local treatments, such as TACE+RFA, have led to improved prognoses for patients with HCC. Ongoing trials combining RFA with radiotherapy are anticipated to yield positive outcomes soon. As per a multidisciplinary consensus document, the choice of local treatment should be determined by the anatomical location of CRLM, which can influence the future utilization of RFA in conjunction with local therapy.^284^ As we gradually uncover the mechanism by which iRFA leads to tumor progression, targeted drugs and immunotherapy have become popular as adjuvant therapies for RFA in studies on HCC and CRLM. In patients with both HCC and CRLM, the application of activated CIK cells after RFA has been shown to improve prognosis.^253^, ^285^ This provides additional options for treating these conditions and demonstrates that cell immunotherapy is a promising avenue for exploration. As antiangiogenic combined immunotherapy has become the first‐line treatment for HCC, further investigation is needed to determine whether combining it with RFA can result in more effective therapeutic outcome. Generally, combination therapy offers promising prospects for liver tumors treatment. Therefore, by addressing the concerns associated with iRFA diagnosis and treatment, this straightforward and minimally invasive approach with precise tumor reduction capabilities will demonstrate a broader range of applications and increased therapeutic value. Moreover, the actual therapeutic impact of RFA depend on the operator. Therefore, future studies should also concentrate on optimizing and standardizing RFA procedures to improve efficacy.

## AUTHOR CONTRIBUTIONS

Jianhua Wu, Wangjun Liao, and Na Huang contributed to the conception, writing, and discussion of this manuscript. Zhiyuan Zhou and Yuanwen Huang equally contributed and wrote the initial draft of the manuscript. Xinyue Deng, Siting Zheng, Shangwen He, Genjie Huang, Binghui Hu, and Min Shi contributed to the initial draft of the manuscript. All authors have approved the final version of the manuscript.

## CONFLICT OF INTEREST STATEMENT

The authors declare that they have no conflict of interest.

## ETHICS STATEMENT AND CONSENT TO PARTICIPATE

Not applicable.

## Data Availability

Not applicable.
